# Altered mucosal bacteria and metabolomics in patients with Peutz–Jeghers syndrome

**DOI:** 10.1186/s13099-024-00617-9

**Published:** 2024-04-27

**Authors:** Sui Wang, Guan-Jun Kou, Xiao-Han Zhao, Gang Huang, Jue-Xin Wang, Lin Tian, Xiu-Li Zuo, Yan-Qing Li, Jia-Yong Wang, Yan-Bo Yu

**Affiliations:** 1 Department of Gastroenterology, Qilu Hospital of Shandong University, 107 Wenhuaxi Road, Jinan, 250012 Shandong People’s Republic of China; 2 Department of Respiratory Medicine, The Second Hospital of Shandong University, Jinan, 250033 Shandong People’s Republic of China; 3 Department of General Surgery, Qilu Hospital of Shandong University, Jinan, 250012 Shandong People’s Republic of China

**Keywords:** Peutz–Jeghers syndrome, Metabolomics, LC‒MS, Mucosa-associated microbiota, Bacteria

## Abstract

**Background:**

Peutz–Jeghers syndrome (PJS) is a rare genetic disorder characterized by the development of pigmented spots, gastrointestinal polyps and increased susceptibility to cancers. Currently, most studies have investigated intestinal microbiota through fecal microbiota, and there are few reports about mucosa-associated microbiota. It remains valuable to search for the key intestinal microbiota or abnormal metabolic pathways linked to PJS.

**Aim:**

This study aimed to assess the structure and composition of mucosa-associated microbiota in patients with PJS and to explore the potential influence of intestinal microbiota disorders and metabolite changes on PJS.

**Methods:**

The bacterial composition was analyzed in 13 PJS patients and 12 controls using 16S rRNA gene sequencing (Illumina MiSeq) for bacteria. Differential analyses of the intestinal microbiota were performed from the phylum to species level. Liquid chromatography-tandem mass spectrometry (LC‒MS) was used to detect the differentially abundant metabolites of PJS patients and controls to identify different metabolites and metabolic biomarkers of small intestinal mucosa samples.

**Results:**

High-throughput sequencing confirmed the special characteristics and biodiversity of the mucosa microflora in patients with PJS. They had lower bacterial biodiversity than controls. The abundance of intestinal mucosal microflora was significantly lower than that of fecal microflora. In addition, lipid metabolism, amino acid metabolism, carbohydrate metabolism, nucleotide metabolism and other pathways were significantly different from those of controls, which were associated with the development of the enteric nervous system, intestinal inflammation and development of tumors.

**Conclusion:**

This is the first report on the mucosa-associated microbiota and metabolite profile of subjects with PJS, which may be meaningful to provide a structural basis for further research on intestinal microecology in PJS.

**Supplementary Information:**

The online version contains supplementary material available at 10.1186/s13099-024-00617-9.

## Introduction

Peutz‒Jeghers syndrome (PJS) is a rare genetic disease characterized by pigmentation of the skin and mucosa, multiple hamartomatous polyps of the gastrointestinal tract, and increased susceptibility to multisystem cancer [1–3]. In addition to heredity, the intestinal microenvironment is the most important and complex microbial dynamic system in the human gut, and its changes and associated metabolites may have an impact on PJS. At present, it is not clear whether the imbalance of intestinal microbiota is related to PJS; however, intestinal microbiota is a new focus of current research, and it plays a crucial role in maintaining a dynamic balance [4]. A number of studies have shown that intestinal bacteria have various important physiological functions, including immune regulation, metabolism, nervous system regulation, enteric fermentation, vitamin biosynthesis [5–8]. The total surface of the gastrointestinal tract is approximately 250–400 square meters, and the appropriate temperature and abundant nutrients in the intestine are conducive to the colonization of diverse microorganisms [9, 10]. At present, extensive studies on fecal flora have proven that intestinal dysbiosis can cause various diseases [11–17]. In previous studies, we analyzed the characteristics of fungal and bacterial microbial community structure in the feces of PJS patients and confirmed that PJS patients have significant intestinal microbial characteristics represented by feces, and the intestinal microbial diversity of PJS patients is significantly reduced. Potential biomarkers in fecal samples of PJS were identified. The differences in the composition and physiological effects of the intestinal mucosal surface and lumen microecological environment have been confirmed and need further exploration [18]. Intestinal microbiota includes not only the luminal microbiota represented by fecal flora but also mucosa-associated microbiota. The flora adheres to the intestinal mucosa to form a biological barrier of the intestinal mucosa, which protects the intestinal epithelium from pathogenic bacteria, resists colonization of pathogenic bacteria and maintains normal physiological functions of the intestine. Li et al. and Choo et al. proved that there were significant differences in the composition of microflora in colorectal mucosa and stool in patients with colorectal cancer [19] and irritable bowel syndrome (IBS) [20]. Some studies have shown that the mucosa-associated microbiota may play a more important role in regulating the host immune system and causing some chronic inflammatory diseases, and their composition changes and metabolic activities may have an impact on the physiological health and disease status of the host [21–24]. Furthermore, fecal flora is frequently mixed with food and oral bacteria [4], The use of fecal samples as representative microbiome specimens can result in inaccurate data due to issues during collection, transportation, and preservation. Mucosal bacteria attached to the intestinal mucosa are more resistant to antibiotics than luminal bacteria without growing in biofilms [21]. It is difficult to change the structure and composition of mucosa-associated microbiota with stronger stability [21, 25]. In some well-studied diseases such as IBD, IBS or CRC, more and more evidence showed that the signature of ‘mucosa-associated microbiota’ differs from fecal or luminal microbiota and has the potential to be used to distinguish between diseased and healthy status [26]. Intestinal microorganisms can produce small molecules through various metabolic pathways, thus affecting intestinal inflammation and pathophysiology [27–29]. However, the changes in intestinal mucosal metabolites in PJS patients are still unclear. Currently, liquid chromatography-tandem mass spectrometry (LC‒MS) has been widely used to analyze disease metabolism and screen biomarkers [30–32]. In this study, we further analyzed metabolites in intestinal mucosal samples of PJS patients. Due to the higher difficulty of collecting samples and the lower density of bacteria on the small intestine, there are still few studies on small intestinal mucosal microbiota and mucosal metabolites in the world. While the cohort was limited in size, this is the first study to picture intestinal mucosa-associated microbiota and metabolites of PJS patients, and this study could provide a research basis for exploring the possible disease-related metabolic pathways of PJS.

## Materials and methods

### Study population

In this study, all recruited individuals were from the Digestive Endoscopy Center of Qilu Hospital, Shandong University, from October 2021 to October 2022, and the control group included patients who underwent colon surgery due to trauma and colonic multiple diverticula during the same period. To reduce the influence of age, diet, environment and living habits on intestinal microbiota [33–36], all the subjects were Han people living in Shandong Province for a long time. A total of 25 subjects were included in the study, including 13 PJS patients (Group P) and 12 non-PJS controls (Group H).

The diagnostic criteria for PJS are individuals with (1) a positive family history of PJS and any number of histologically confirmed PJS polyps or characteristic, prominent, mucocutaneous pigmentation or (2) a negative family history of PJS and ≥ 3 histologically confirmed PJS polyps or any number of histologically confirmed PJS polyps and characteristic pigmentation were confirmed as having PJS [37].

Inclusion criteria of control group: (1) Age: 18–80 years old, no gender restrictions, body mass index < 30; 2) Individuals who underwent colon surgery due to trauma and colonic multiple diverticula during the same period; (3) Patients in generally good condition with relatively stable vital signs; (4) No special diet; (5) No history of intestinal disorders and endoscopic or histological signs before biopsy.

Individuals were excluded if they had cardiopulmonary insufficiency, liver and kidney insufficiency, ascites, jaundice, cirrhosis, coagulation disorders, pregnancy, breastfeeding, acute gastrointestinal bleeding, fever, colorectal malignancies and postoperative colorectal malignancies, and other patients who were not suitable for colonoscopy, were unable to cooperate, were orally taking medicines (such as antibiotics, probiotics or prebiotics) that may affect the gut microbiota within the last 2 weeks, and patients who could not provide valid information.

The program and feasibility of the application of human mucosal specimens in this study were reviewed and approved by the Ethics Committee of Qilu Hospital of Shandong University before implementation (IRB number: KYLL-202011-081-2).

### Fecal sample collection

All subjects underwent intestinal preparation the day before the examination, and the color and character of the stool were continuously observed until colorless and slag-free watery stool appeared, which was feasible for colonoscopy. Before preparing for colonoscopy, patients were fully informed of the purpose and specific process of the study. For PJS patients, the prebiopsy site was repeatedly rinsed with normal saline under double balloon enteroscopy, and the resected small intestinal polyps were immediately placed in a specimen bag after suction with negative pressure, frozen in liquid nitrogen, and transported to a refrigerator at − 80 °C for storage. In the healthy control group, a portion of marginal small intestinal tissue approximately the size of two soybean grains (the weight was at least 4 g) was cut from the intestinal tissue removed during total colectomy, immediately placed in a cryostorage tube, and then transported in liquid nitrogen to a refrigerator at − 80 °C for storage.

### DNA extraction

The samples were shipped to Major-Bio (Shanghai, China) for high-throughput sequencing. The E.Z.N.A.® soil DNA Kit (Omega Bio-Tek, Norcross, GA, U.S.) was used to extract microbial DNA from all samples according to the manufacturer’s protocol. The final DNA concentration and purification were determined using a NanoDrop 2000 UV‒vis spectrophotometer (Thermo Scientific, Wilmington, USA), and the DNA quality was determined using 1% agarose gel electrophoresis. Polymerase chain reaction (PCR) (ABI Gene Amp 9700, ABI, USA) was used to amplify the V3-V4 region of the bacterial 16S rRNA gene using primers 338F (ACTCCT ACGGGAGGCAGCAG) and 806R (ACTCCTACGGGA GGCAGCAG) and Trans Start Fast pfu DNA polymerase (Trans Gen, Beijing, China). The resulting PCR products were extracted from a 2% agarose gel, further purified using the AxyPrep DNA Gel Extraction Kit (Axygen Biosciences, Union City, CA, USA), and quantified using QuantiFluor™-ST (Promega, USA). Purified amplicons were pooled in equimolar and paired-end sequenced on an Illumina MiSeq PE300platform (Illumina, USA) according to the standard protocols by Majorbio Bio-Pharm Technology Co. Ltd. (Shanghai, China). The raw reads were submitted to the NCBI Sequence Read Archive (SRA) database with accession numbers: PRJNA1091066.

### Processing of sequencing data

The raw 16S rRNA gene sequencing reads were demultiplexed, quality-filtered by fastp and merged by FLASH, the 300 bp reads were truncated at any site receiving an average quality score (< 20) over a 50 bp sliding window, and the truncated reads < 50 bp were discarded (2) overlapping sequences > 10 bp were assembled according to overlapped sequence. The max mismatch ratio of overlap region is 0.2. Reads that could not be assembled were discarded; (3) Samples were distinguished according to the barcode and primers, and the sequence direction was adjusted, exact barcode matching, 2 nucleotide mis-match in primer matching. Operational taxonomic units (OTUs) with 97% similarity cut off were clustered using UPARSE, and chimeric sequences were identified and removed. The taxonomy of each OTU representative sequence was analyzed by RDP Classifier against the 16S rRNA database (Silva v138) using confidence threshold of 0.7.

### Sample preparation and quality control for LC‒MS metabolomics

Each 50 mg mucosa sample were mixed with 1 mL water–methanol-acetonitrile (1:2:2(v:v)), vortexed for 30 s, homogenized at 45 Hz for 4 min, sonicated 5 min at 4 °C, and then incubation 1 h at − 20 °C. The mixtures were centrifuged at 12,000 rpm for 10 min at 4 °C, then the supernatant was transferred to UHPLC-QE Orbitrap/MS analysis. UHPLC system (1290, Agilent Technologies) with a UPLC HSS T3 column (2.1 mm × 100 mm, 1.7 um) coupled to Q Exactive Orbitrap (Thermo Fisher Scientific) were used. The formic acid (0.1%) and ammonium acetate (5 mM) were the solvent A for positive (ES +) and negative (ES−), respectively. Solvent B was the acetonitrile. About 2 μL sample were injected at 4 °C for analysis. The gradient elution of solvent B as follows: 1%, 0–1 min; 99%, 8 min;99%, 10 min; 1%, 10 min; 1%, 12 min. The thermo Q Exactive Orbitrap mass spectrometer can control the Xcalibur version 4.0.27 to acquire the full scan survey MS and MS/MS spectra. The spray voltage of ES + was 3.8 kV, and 3.1 kV for ES−.

The raw data were converted into mzML format using ProteoWizard (http://proteowizard.sourceforge.net/), and preprocessed with R package XCMS v3.2.78 The processed data include peak intensity, mass-to-charge ration (m/z), and retention time (RT). The metabolites were identified with the featured peaks according to the software OSI/SMMS version 1.0. Impurity peaks and duplicate identifications were eliminated. For each data set, we removed the compounds that were present in fewer than 50% of samples within a study. The identification of tentative metabolite was mapped in MS and MS/MS database using the The Human Metabolome Database (HMDB) (https://hmdb.ca). If some peak both mapped in MS and MS/MS database, MS/MS data was the result. Unmapped was defined as MS or MS/MS neither mapped. Metabolites of all samples of equal volume were mixed to prepare quality control samples (QC). In the process of instrumental analysis, one QC sample was inserted into every eight samples to investigate the repeatability of the entire analysis process.

### Data preprocessing and database

Baseline filtering, peak identification, integration, retention time correction, and peak alignment were completed through Progenesis QI (Waters Corporation, Milford, USA). Finally, the data matrix of retention time, mass–charge ratio and peak intensity was obtained. Data analysis was performed on the Majorbio Cloud Platform (https://cloud.majorbio.com) to upload data for subsequent analysis, data pretreatment, removing QC samples Relative standard deviation (Relative standard deviation, RSD) > 30% of the variables, and log10 logization process, to obtain the final data matrix for subsequent analysis.

### Statistical analyses

Mothur software (version v.1.30.2 https://mothur.org/wiki/calculators/) was used to calculate alpha diversity, and the Wilcoxon rank sum test was used to analyze the difference between groups. PCoA analysis based on the Bray‒Curtis distance algorithm was used to check the similarity of microbial community structure among samples, and combined with Linear discriminant analysis of effect sizes (LEfSe) (http://huttenhower.sph.harvard.edu/galaxy/root?tool_id=lefse_upload), bacterial groups with significant differences in phylum to species abundance between different groups were identified(LDA = 2). A P of < 0.05 was defined as statistically significant. The SPSS statistical package (version 26.0) was used to analyze the data.

## Result

### Clinical baseline data

A total of 25 subjects were included in the study, including 13 PJS patients (Group P) and 12 non-PJS controls (Group H). The characteristics of the subjects are shown in Table 1. The mean age of the 13 PJS patients (P group) was 26.08 ± 8.45 years, including 6 females (46.15%). The control group (Group H) included 12 patients, with an average age of 31.83 ± 8.21 years old, of whom 4 were female (33.33%). Detailed information on the subjects in this study is shown in Table 1. No statistically significant differences in age, sex or body mass index (BMI) were observed between the groups (P > 0.05).

**Table 1:** The clinical data of patients with Peutz–Jeghers syndrome (PJS) and controls

	Group P (n = 13)	Group H (n = 12)	*P*
Gender (%female)	6:7 (46.15%)	4:8 (33.33%)	0.317
Age (min–max)	26.077 ± 8.451 (14–36)	31.833 ± 8.211 (17–47)	0.098
BMI (min–max)	22.54 ± 4.57 (17.31–35.76)	22.58 ± 3.45 (16.61–29.05)	0.854

A P of < 0.05 was defined as statistically significant

### Analysis of mucosa-associated bacterial composition

**Fig. 1:**
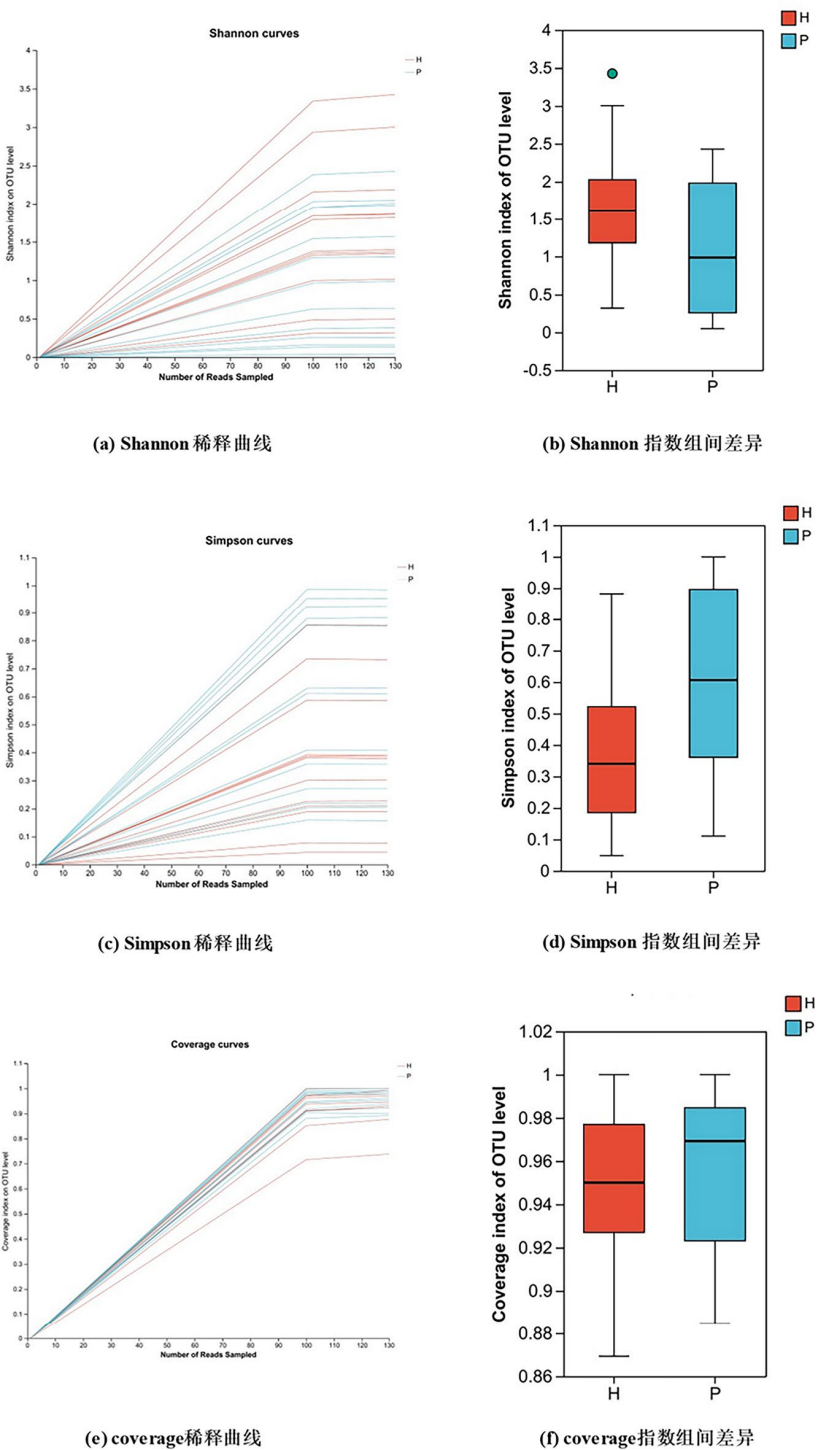
Alpha diversity of the mucosa-associated bacteria in different groups at the OTU level. Rarefaction curve indicated that the sample was sufficient. The Simpson index is higher in Group P than Group H. However, Shannon (Student’s test, P = 0.05137), Simpson (Student’s test, P = 0.06055), Coverage (Student’s test, P = 0.4943) and other index analyses did not reach statistical significance. (P: PJS patient group; H: healthy control group)

As shown in Fig. 1, the Rarefaction curve gradually flattened with increasing sequencing volume, indicating that the sample was sufficient, and the result could represent the small intestinal mucosa microbiome. The alpha diversity was estimated through different indices. The Shannon Diversity Index (sometimes called the Shannon–Wiener Index) is a way to measure the diversity of species in a community. The higher the value, the higher the diversity of species in a particular community. Simpson’s Diversity Index is another way to measure the diversity of species in a community. The value for Simpson’s Diversity Index ranges between 0 and 1. The higher the value, the lower the diversity, and the Coverage index is usually used to observe the Good’s coverage at the OTU level. As shown in Fig. 1, the microbial community diversity of Group P was lower than that of Group H. However, Shannon (Student’s test, P = 0.05137), Simpson (Student’s test, P = 0.06055) and other index analyses did not reach statistical significance. The rank-abundance curve is another way of analyzing diversity, explaining it in two ways: the abundance of the population and the evenness of the community. The steeper decline curve of Group P compared with Group H indicates a higher proportion of dominant bacteria in the samples of Group P and lower species diversity (Additional file 1: Fig. S1).

### Analysis of Venn diagram

**Fig. 2:**
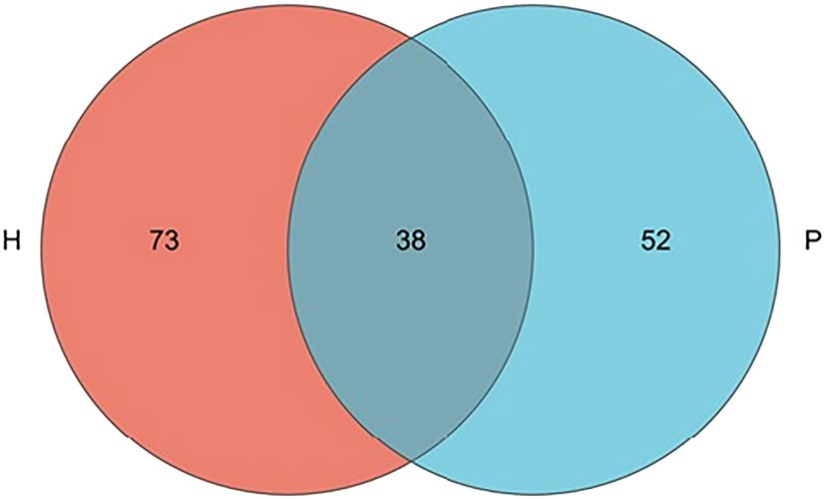
Venn diagram. In the Venn diagram, rare microbial OTUs were removed without subsampling. There was much overlap in the OTUs of each group. Group P: Peutz‒Jeghers syndrome (PJS) patients; Group H: non-PJS controls

A total of 897,725 high-quality sequences were detected from the 25 samples. The intestinal microbiota in all samples were classified into 13 phyla, 24 classes, 48 orders, 80 families, 123 genera, 142 species, and 164 OTUs. To analyze the overlap of intestinal mucosal-associated microbiota composition between PJS patients and controls, a Venn diagram is shown in Fig. 2. The number of OTUs in Group P was 90, the number of OTUs in Group H was 111, and the common number of OTUs in the two groups was 38. There were 52 unique OTUs in Group P and 73 unique OTUs in Group H.

### Differences in bacterial abundance between groups

**Fig. 3:**
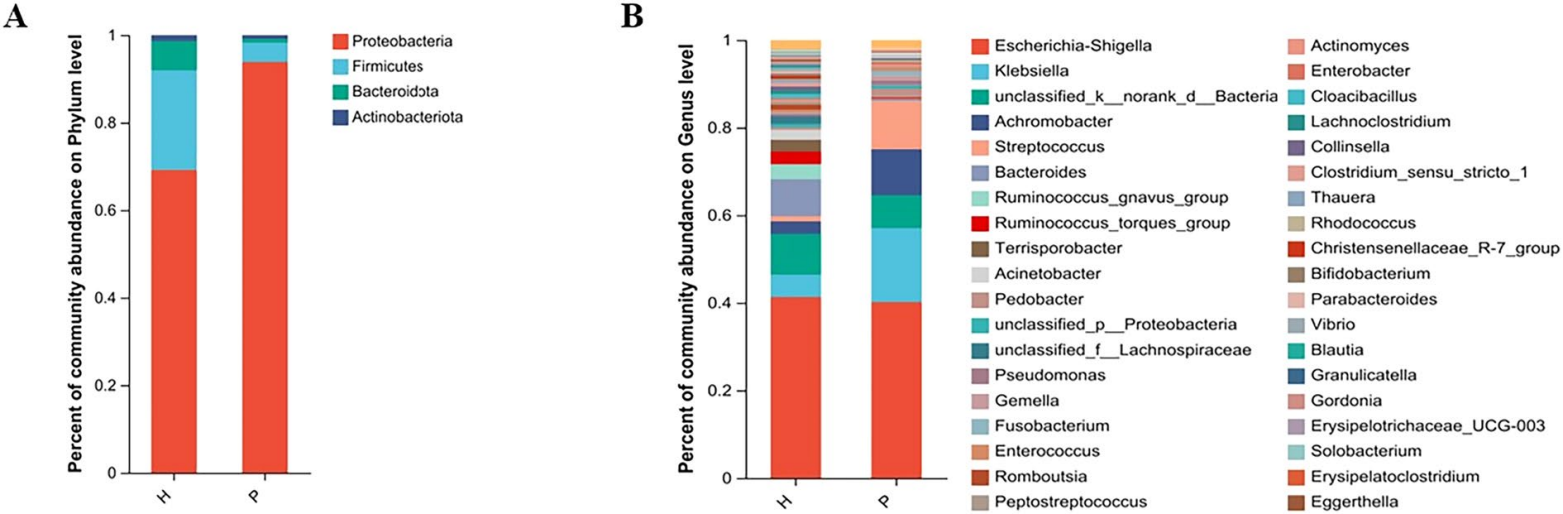
Community bar plot analysis of bacteria at the phylum and genus levels. The vertical coordinate in the figure represents the proportion of species in the samples of each group, and the species are represented by different colors in the figure (H: healthy control group; P: PJS patient group)

Proteobacteria, Firmicutes, Bacteroidetes and Actinobacteriota constituted the four main phyla of mucosal bacteria (Fig. 3A). The proportion of Proteobacteria in Group P (93.86%) was significantly higher than that in Group H (69.20%), while the proportions of Firmicutes, Bacteroidetes and Actinobacteria were significantly reduced in Group P compared to Group H. As shown in Fig. 3B, at the genus level, *Escherichia-Shigella* (40.24%) and *Klebsiella* (16.80) accounted for the highest proportion in Group P, and the abundance of *Klebsiella* (16.80%), *Achromobacter* (10.47%) and *Streptococcus* (11.07%) in Group P was significantly higher than that of *Klebsiella* (5.0%), *Achromobacter* (2.88%) and *Streptococcus* (1.22%) in Group H, while the abundance of other genera was decreased.

### Comparative analysis of different species between groups

**Fig. 4:**
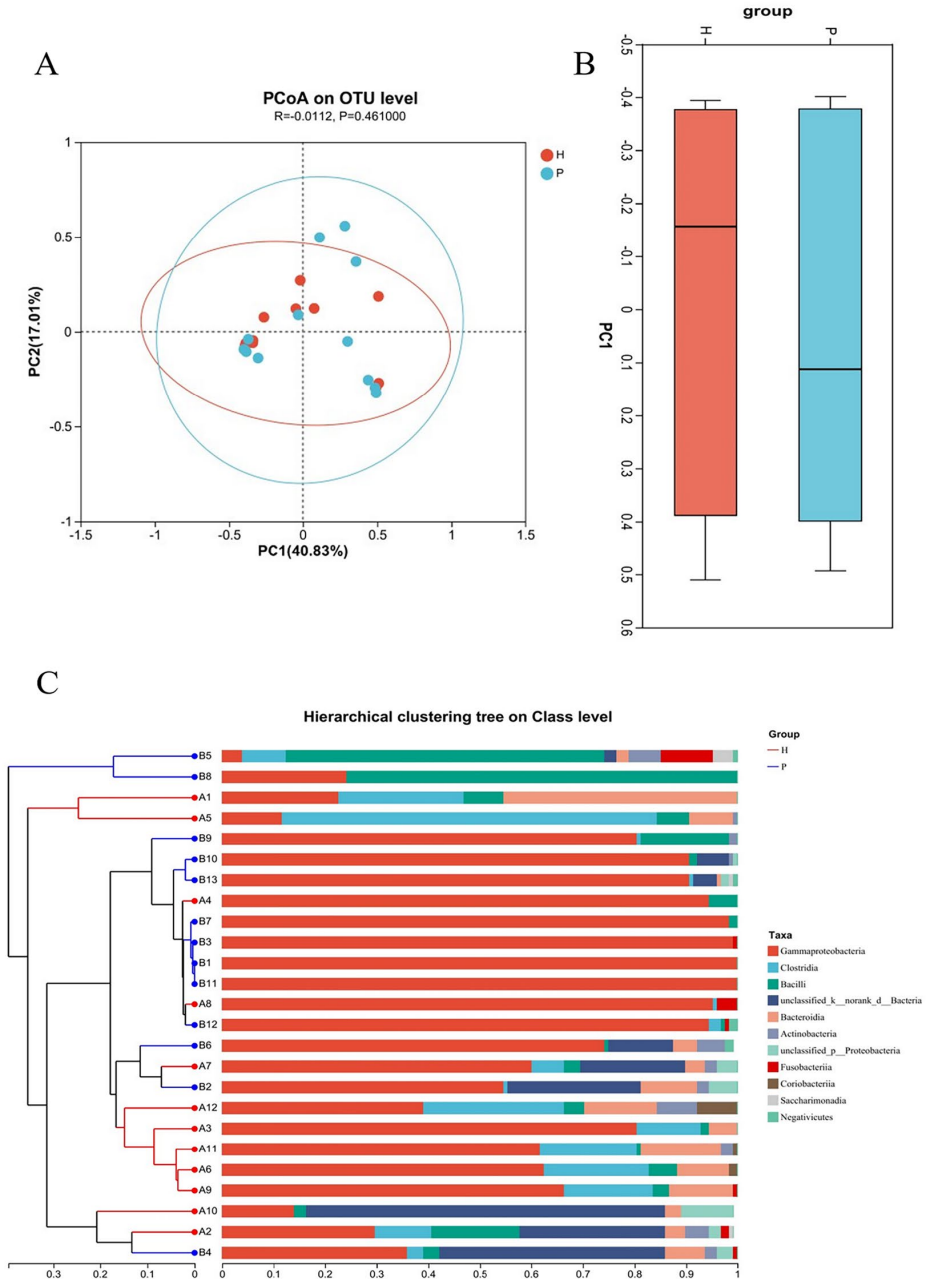
Beta diversity of mucosa-associated bacteria. **A** The group ellipses of the 95% confidence interval overlapped significantly, which could not effectively distinguish the difference between groups (P = 0.4610), and the samples in the corresponding group of PJS patients were more discrete. **B** Based on OTU level analysis, the difference between groups was smaller than the difference within groups. **C** Bar graph of microflora relative abundance and hierarchical clustering of evolutionary relationships at the class level, revealing 5 clusters in the community branches of the two groups; (H: healthy control group, P: PJS patient group)

PCoA was used to analyze beta diversity in the community composition of the two groups. Figure 4 shows that the difference between Group H and Group P at the class OTU was less significant than the difference within the groups (P = 0.4610). Based on the Bray–Curtis distance clustering analysis, no obvious intergroup clustering was found. There was no obvious evolutionary classification relationship between samples, the samples of patients and controls were mixed, and the overlap of the 95% confidence ellipse was obvious. The samples within Group P were more discrete, suggesting that the mucosal bacterial community heterogeneity of PJS patients was more obvious than that of healthy people. Hierarchical clustering analysis was carried out based on the beta diversity distance matrix, and the clustering tree was constructed by UPGMA (Unweighted Pair-group Method with Arithmetic Mean) algorithm. The degree of similarity or difference in bacterial community composition was visualized. The UPGMA clustering tree in class level was generated in Fig. 4C, which showed that 5 clusters in 2 groups, indicating that the bacterial community composition was significantly different and clustered dividedly.

### Differences in species abundance between groups

At the family level, the relative abundances of *Oscillospiraceae*, *Eggerthellaceae*, *Sutterellaceae* and *Butyricicoccaceae* in controls were significantly higher than those in PJS patients. In the intestinal mucosal microbiota of PJS patients, the relative abundance of *Xanthobacteraceae* was significantly higher than that of controls. At the species level, the relative abundances of *unclassified_g_Bacteroides, unclassified_f_Lachnospiraceae, Erysipelatoclostridium_ramosum, unclassified_g. _Romboutsia, metagenome_g_Blautia* and other strains in controls were significantly higher than those in PJS patients (the results are shown in Additional file 2: Fig. S2).

### Potential biomarkers in patients with PJS

**Fig. 5:**
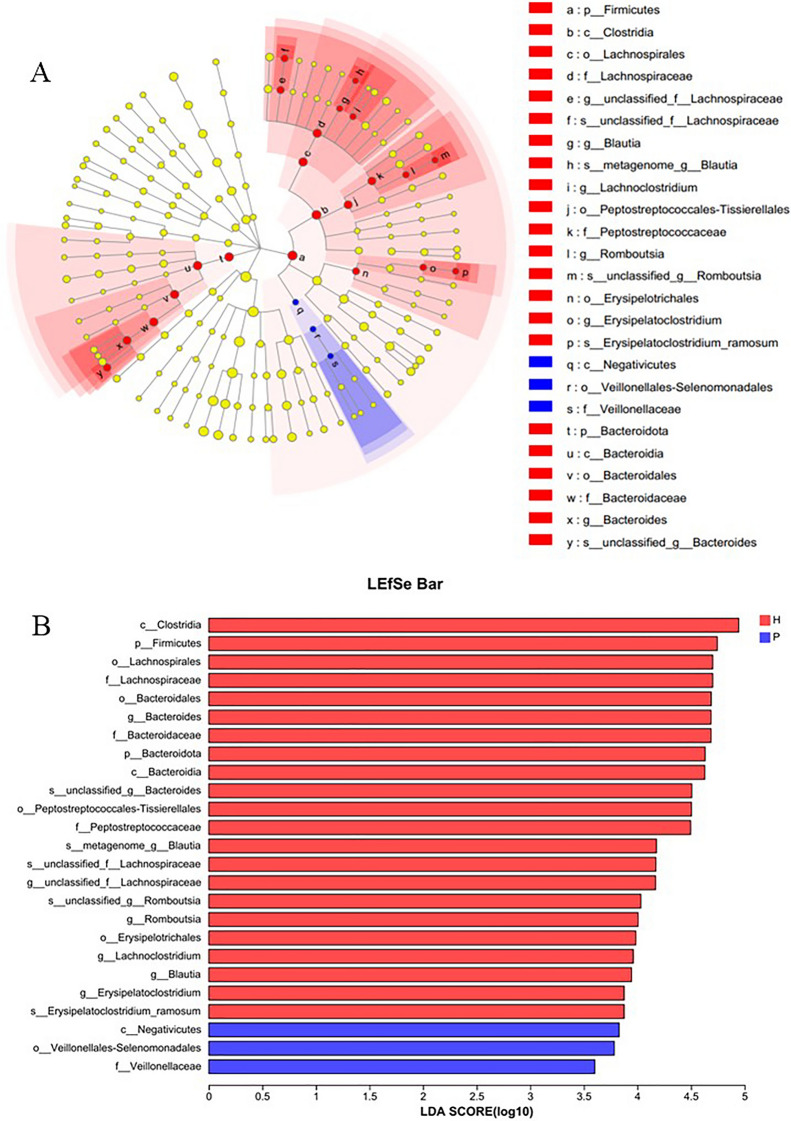
Linear discriminant analysis of effect sizes (LEfSe) bar plot. The yellow dots in **A** represent species with no significant difference between the two groups, and the diameter is proportional to the LDA; the length of the bar in **B** is the influence of the different species; red represents the healthy control group, and blue represents the PJS patient group (LDA = 2.0)

Potential biomarkers were screened by LEfSe. As shown in Fig. 5, the taxa that have the main influence on the species difference between the two groups at various levels. In Group H, the significantly enriched phyla included Firmicutes and Bacteroidota. At the class level, *Clostridia* and *Bacteroidia* significantly enriched. At the order level, *Bacteroidales*, *Lachnospirales*, *Peptostreptococcales*-*Tissierellales*, *Erysipelotrichales* significantly enriched. On the family level, *Bacteroidaceae*, *Lachnospiraceae* and *Peptostreptococcaceae* significantly enriched. At the genus level, significantly enriched genus included *Blautia, Lachnoclostridium, Romboutsia, Bacteroides,* etc. The key distinguishing units in the mucosal microbiota of Group P were *Negativicutes* (class), *Veillonellales*-*Selenomonadales* (order), and *Veillonellaceae* (family level).

### Data standardization and Partial least squares-discriminant analysis (PLS-DA)

**Fig. 6:**
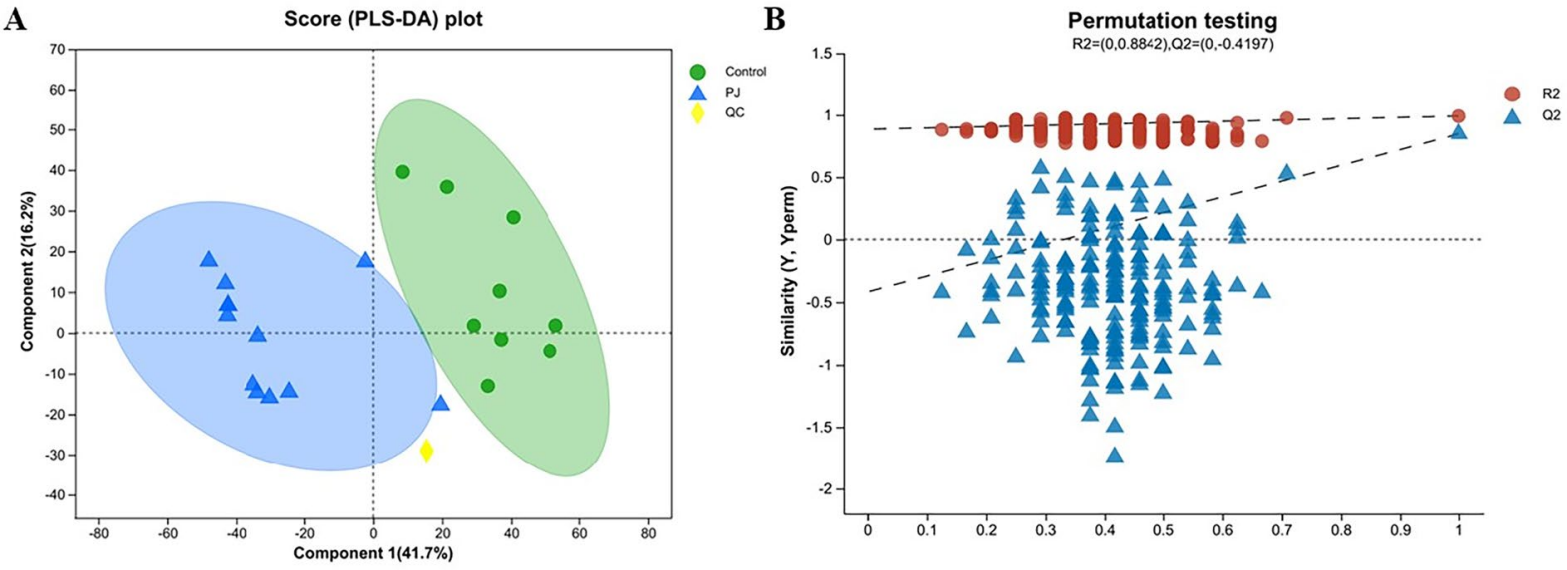
PLS-DA plot. **A** The separation between the two groups is obvious, the distance within the samples is close, and the classification effect is significant. **B** The two dotted lines represent regression lines of R2 and Q2. R2 is (0, 0.8842) and Q2 is (0, − 0.4197). The intercept between the regression line of Q2 and the Y-axis is less than 0, which means that the research model is robust

PLS-DA analysis is shown in Fig. 6. The horizontal coordinate is the permutation retention degree, in the same order as the original Model Y variable. Points with permutation retention degree 1 are the values of R2 and Q2 of the original model, and the vertical coordinate is the values of the permutation test of R2 and Q2. The intercept of the Q2 regression line and Y axis was the evaluation standard of the displacement test, and an intercept < 0 indicated that the research model was reliable and that there was no overfitting phenomenon. The distribution of RSD is shown in Additional file 3: Fig. S3.

### Differentially abundant metabolite analysis

Differentially abundant metabolites were screened, and visual analysis was carried out through the differential volcano map (the result is shown in Additional file 4: Fig. S4). The dots in the figure represent a metabolite, the red dots represent metabolites with upregulated expression, the blue dots represent metabolites with downregulated expression, and the closer to the left and right sides and the upper points, the more significant the differential expression, while the gray dots represent products with no significant differences in metabolism. In this analysis, the number of cationic peaks meeting all screening conditions was 1590, among which the number of differentially abundant metabolites with identified names was 532, while the number of anion peaks meeting the differential screening conditions was 1871, and the number of differentially abundant metabolites with identified names was 405.

### The analysis of variables important in the projection

**Fig. 7:**
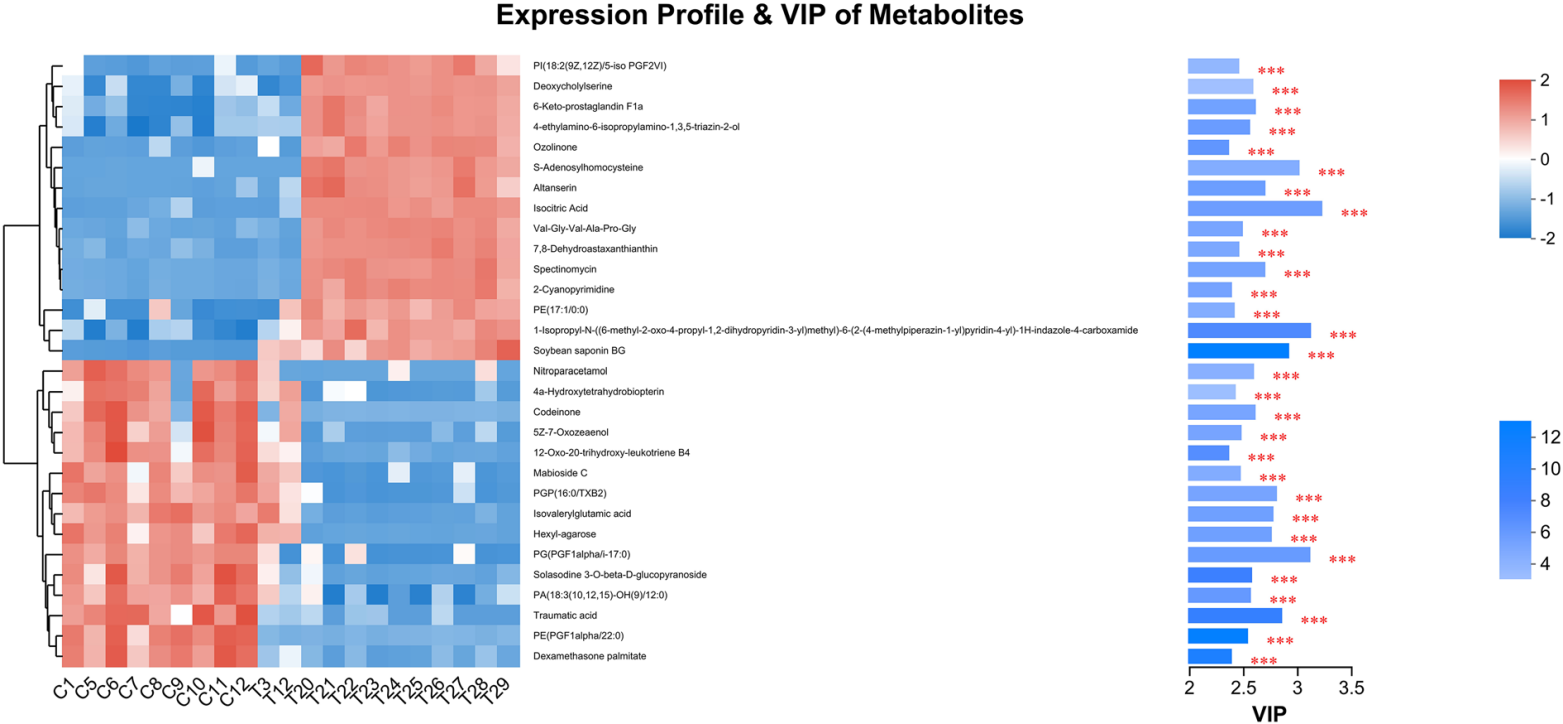
Metabolite clustering tree and VIP bar plot. Each column represents a sample, and each row represents a metabolite. The denser the branch of the metabolite clustering tree, the more similar the expression patterns of all metabolites in the sample. In the histogram of VIP metabolites, the length of the blue bar indicates the contribution of metabolites to the difference between the two groups, the color of the VIP bar indicates the significance of the difference between the two groups of metabolites, and the lower the P value is, the darker the color (*P < 0.05, **P < 0.01, ***P < 0.001)

Figure 7 shows the heatmaps and variable importance in projection (VIP) of metabolite clustering. Metabolites significantly upregulated in Group P included deoxycholine serine, 6-ketoprostaglandin F1a, 4-ethylamino-6-isopropyl amino-1,3,5-triazine-2-ol, ozone, S-adenosine homocysteine, Altanserin, isocitric acid, Val-Gly-Val-Ala-Pro-Gly, 7,8-dehydrotaurine, spectinomycin, 2-cyanopyrimidine, and soybean saponin. Significantly downregulated metabolites included nitroparacetamol, 4a-hydroxy-tetrahydrobiotrexate, codeinone, 5Z-7-oxazolenol, 12-oxo-20-trihydroxyleukotriene B4, maphioside C, isoamylglutamate, hexylagarose, traumatic acid, and dexamethasone palmitate.

### KEGG metabolic functional pathway

**Fig. 8:**
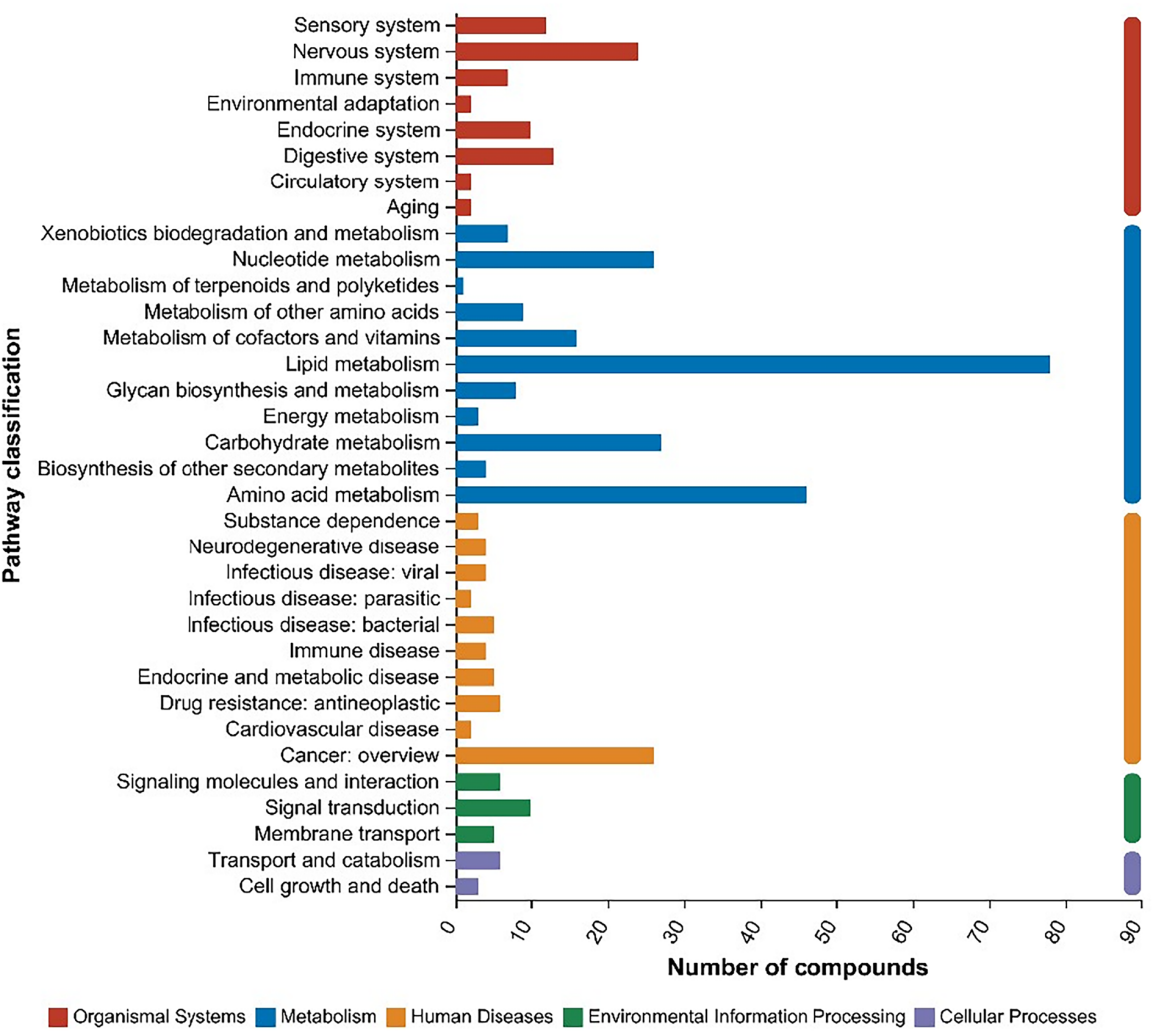
Bar chart of KEGG functional pathway metabolism. The vertical coordinate is the secondary KEGG metabolic pathway, and the horizontal coordinate is the number of metabolites annotated to this pathway. Colors such as blue, red, yellow, green and purple represent the primary classification of the 5 major metabolic functional pathways in this study. They are metabolism, organismal systems, human diseases, environmental information processing and cellular processes

The KEGG metabolic function pathway can be divided into seven major pathways: metabolism, genetic information processing, environmental information processing, cellular processes, organismal systems, human diseases, and drug development. As shown in Fig. 8, the primary classification of major metabolic pathways in mucosa mainly includes metabolism, organismal systems, human diseases, environmental information processing and cellular processes. Regarding secondary metabolites, metabolites in biosynthetic pathways, including lipid metabolism, amino acid metabolism, carbohydrate metabolism, nucleotide metabolism, nervous system, cofactor and vitamin metabolism and other secondary metabolites, were significantly upregulated.

### Enrichment analysis of KEGG pathways

**Fig. 9:**
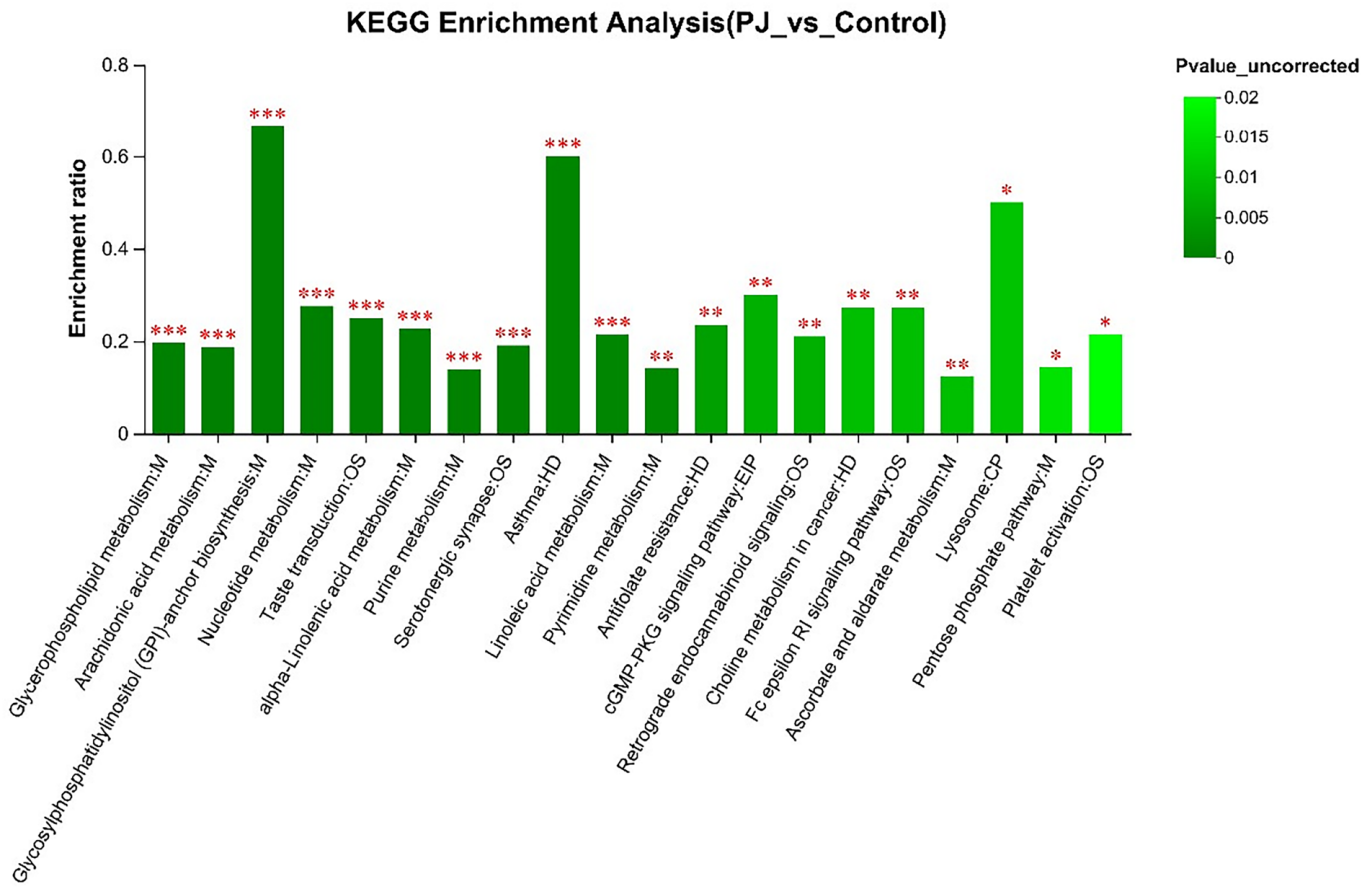
KEGG pathway enrichment analysis. The P value is represented by the change in the color gradient on the right, the horizontal coordinate is the metabolic pathway name, and the vertical coordinate is the enrichment ratio (*P < 0.05, **P < 0.01, ***P < 0.001)

As shown in Fig. 9, 10 metabolic pathways showed lower P values and higher pathway enrichment rates between Groups P and H, including glycosylphosphatidylinositol (GPI)-anchored protein biosynthesis, asthma, pyrimidine metabolism, taste transduction, 5-hydroxytryptamergic synapse, linoleic acid, α-linolenic acid metabolism, purine metabolism, arachidonic acid metabolism, and glycerophospholipid metabolism, suggesting that these metabolic pathways may play a special role in the clinical manifestations of PJS patients.

## Discussion

Studies have shown that the changes in bacterial communities in the intestinal lumen and intestinal mucosa are different in patients with IBS, inflammatory bowel disease (IBD), and colorectal cancer (CRC) [38–40], indicating that there are important differences between the intestinal luminal microenvironment and intestinal mucosal microenvironment [41]. The fecal microbiota is more sensitive and susceptible to the intestinal environment, diet, stress and other factors, while the intestinal mucosal microbiota is less affected by intestinal lumen contents and other environmental conditions.

In this study, the intestinal mucosal microbiota was analyzed, and the OTUs were clustered according to 97% 16S rRNA gene sequence similarity. A total of 164 OTUs were obtained in 25 samples, which was significantly reduced compared with the total number of OTUs obtained in the fecal microbiota of PJS patients [42], indicating that the diversity of mucosal microbiota was less than that of luminal microbiota. More than 90% of the mucosal bacteria are anaerobic, and the dominant bacteria include *Bacteroides, Eubacterium, Bifidobacterium, Fusobacterium, Bacteroides,* and *Peptostreptococcus* et al. [43]. The diversity of mucosal bacteria in PJS patients decreased, but there was no significant difference in some diversity indices, which may be related to the small sample size. Compared with controls, the samples of PJS patients were more discrete in PCoA, suggesting that the bacterial community of mucosal microflora in PJS patients was more heterogeneous than that in healthy people. Proteobacteria, Firmicutes, Bacteroidetes and Actinobacteriota constituted the main phyla of bacteria in all mucosal samples in the two groups. The proportion of Proteobacteria in the mucosa of PJS patients was significantly higher than that in the mucosa of controls. The main categories of bacterial microbiota were similar to those of fecal microbiota in PJS patients, but the proportion of Proteobacteria increased significantly, suggesting that there was overgrowth of dominant bacterial microbiota different from fecal microbiota. The abundance of *Escherichia-Shigella* and *Klebsiella* was significantly increased in the mucosa of PJS patients, which was confirmed in the fecal microbiota. *Escherichia-Shigella* is associated with mucosal inflammation and IBD pathogenesis [11]. Lipopolysaccharide (LPS) from the *Escherichia-Shigella* genus can activate Toll-like receptor 4 [44] and the nuclear factor kappa B (NF-κB) pathway and induce IL-1, IL-6, and tumor necrosis factor (TNF)-α production [45], causing inflammatory and oxidative damage [46, 47]. *Klebsiella* is often observed in patients with gastrointestinal diseases [48]. These bacteria may play an important role in CRC progression by stimulating epithelial cell proliferation [49]. It is suggested that *Escherichia-Shigella* and *Klebsiella* may have a special role in the pathogenesis of PJS. Some studies have confirmed that Gram-negative bacteria increase significantly in the intestinal mucosa of patients with colon cancer, and intestinal bacteria are closely related to colon tumor tissues, playing an important role in the occurrence and development of colon tumors. Some researchers also believe that it can be used for the early diagnosis and prevention of chronic diseases [50–52]. In this study, we found that the changes in the abundance of Negativicutes and *Veillonellaceae* may have potential value in the diagnosis of PJS, as shown in Fig. 5.

LC‒MS was used to analyze metabolites in intestinal mucosal samples. In this study, microbial communities and metabolic pathways in the mucosa of PJS patients were significantly different, including lipid metabolism, amino acid metabolism, carbohydrate metabolism, nucleotide metabolism, nervous system, cofactor and vitamin metabolism, and other secondary metabolites. It has been proven that lipid metabolism disorder is related to the pathological mechanism of IBS, IBD, CRC and other diseases [39, 53–57]. We found that compared with healthy people, PJS patients have obvious differences in linoleic acid metabolism, α-linolenic acid metabolism, arachidonic acid metabolism, glycerolipid metabolism. Studies have found that linoleic acid plays a positive role in the prevention and treatment of many diseases, such as obesity, cancer, diabetes and cardiovascular diseases [56, 57], and appropriate linoleic acid levels in serum may reduce the risk of cancer. Dysregulation of linoleic acid metabolism exists in a variety of diseases, while dysregulation of choline phospholipid metabolism is generally considered to be associated with cancer, such as breast cancer [58]. Bile acids dissolve lipids and fat-soluble vitamins in the human body and have important physiological functions. Bile acids are synthesized by cholesterol in the liver and released into the duodenum during eating. Approximately 95% of bile acids are reabsorbed in the ileum, and approximately 5% of bile acids are excreted in the feces. Small intestinal polyps and abnormal microbial communities in PJS patients may destroy normal physiological function and affect the bile acid metabolic pathway. An increasing number of studies suggest that bile acids, multifunctional signaling molecules, may affect the central nervous system. Studies have found that a variety of metabolites, such as short-chain fatty acids, bile acids, neurotransmitters and other bioactive substances produced by bacteria in the gut, can participate in the regulation of the central nervous system [59]. In addition, intestinal microbiota can provide lipopolysaccharide, choline, lactic acid and vitamin B for the body to participate in the regulation of the brain–gut axis [60]. The interaction between intestinal microbiota and amino acid metabolism is also crucial for immune homeostasis and regulation of the brain–gut axis. Intestinal microflora participates in the amino acid metabolism pathway and produces the following metabolites: ammonia, biogenic amines, hydrogen sulfide and short-chain fatty acids, including isobutyric acid, isovaleric acid and valeric acid. After the amino acid metabolism pathway is affected, the host may have abnormal energy metabolism, blood glucose regulation ability and inflammatory reactions in the body, such as diabetes, obesity and metabolic syndrome, fatty liver disease and cardiovascular disease [61, 62]. Both *Veillonellaceae* and *Lachnospiraceae* produce short-chain fatty acids through fermentation, which are the main sources of energy for colon cells. *Lachnospiraceae* decreased in PJS patients, the decrease of Lachnospiraceae would reduce butyric acid, an important component of maintaining the intestinal barrier. It can also inhibit the proliferation of intestinal epithelial cells, and butyric acid can also inhibit lipid formation, promote gluconeogenesis in the small intestine, and improve glucose and lipid metabolism. *Veillonellaceae*, which are enriched in PJS patients, is positively associated with acetic acid and propionic acid, instead of butyric acid, which can promote gastrointestinal peristalsis [63, 64]. The abnormal intestinal development in PJS patients may be related to the effects of intestinal metabolic changes on the brain-intestinal axis and intestinal mucosal epithelium. Similar metabolic abnormalities have been found in patients with ulcerative colitis, colon cancer, and breast cancer [65–68]. The above studies suggest that there is a potential association between differences in metabolic pathways and PJS. This study provides a new research idea for the diagnosis and disease control of PJS through LC‒MS technology, but the specific mechanism still needs to be further verified.

This study also has the following limitations: (1) The 16S rRNA gene sequencing provides data on live, dormant, and dead microbes. (2) Our research is an observational study, relying on correlations from relative abundance data, which does not provide information on real abundances in the gut. (3) The site of intestinal mucosal biopsy may also affect the composition of intestinal mucosal microbiota and metabolites. Polyps in PJS patients come from different sites, such as the jejunum and ileum, which may affect the results. (4) The selection of normal controls in this study was based on medical history and clinical diagnosis, and no pathological biopsy of small intestinal mucosa was taken for control. (5) As colonoscopy is rarely performed in healthy physical examination subjects, surgical patients who underwent total colectomy due to emergency trauma or multiple diverticula of the colon were selected as the control group in this study. However, intestinal mucosal microbiota and metabolism may be different under different disease states, and the operative stress state of the control group may also affect the results. (6) We need to adopt newer and safer techniques to collect healthy mucosa from PJ patients and healthy volunteers for more comprehensive analysis. (7) Due to limited techniques, funds and time, the sample size in this study is small, there are few samples with low read numbers, although they had successfully passed the evaluation, the Species level is not reliably achievable with the sequencing approach and the results and conclusions obtained need to be further verified with larger cohort and multiple centers.

The role of the intestinal microflora and abnormal metabolites in PJS pathogenesis remains unknown. In short, this study utilized an LC‒MS metabolomic approach supported by 16S rRNA sequencing to investigate the functional and taxonomical differences and first characterized the intestinal mucosa-associated microbiota and metabolic profile of PJS patients, which may provide a basis for further microecological research on PJS.

### Supplementary Information

**Additional file 1: Figure S1.** Rank-abundance curve. The horizontal coordinate represents the OTU number ranking, the vertical coordinate represents the relative percentage of species, and the horizontal coordinate at the end of the curve extension represents the number of species in the sample (the blue line represents the PJS patient group; the red line represents the healthy control group).

**Additional file 2: Figure S2.**Differences in family and species abundance between groups. Different colors represent different groups, and the X-axis represents the average relative abundance of different groups and the differences between groups at the family and species levels (* P<0.05, ** P<0.01; H: the healthy control group, P: the PJS patient group).

**Additional file 3: Figure S3.** Relative standard deviation (RSD) distribution curve. For the overall data, RSD<0.3, and the cumulative proportion of peaks<70%, suggesting that the quality control data are qualified and can be further analyzed.

**Additional file 4: Figure S4.** Volcanic map of differences between anions and cations. The red dots represent metabolites with upregulated expression, the blue dots represent metabolites with downregulated expression, and the gray dots represent metabolites with no significant differences. The horizontal coordinate is the change value of the difference in the expression of metabolites between the two groups, log 2FC (fold change value); the vertical coordinate is the statistical test value of the difference in the expression of metabolites.

## Data Availability

The authors confirm that sequence data were submitted to the NCBI Sequence Read Archive database with accession numbers: PRJNA1091066.

## Declarations

### Disclaimer

The views expressed in the submitted article are authors’ own and not an official position of the institution or funder.
