# Supplementary figures and images for: Altered mucosal bacteria and metabolomics in patients with Peutz–Jeghers syndrome
Source: Gut Pathog. 2024 Apr 27;16:25. doi: 10.1186/s13099-024-00617-9 (PMC11056063; doi:10.1186/s13099-024-00617-9)

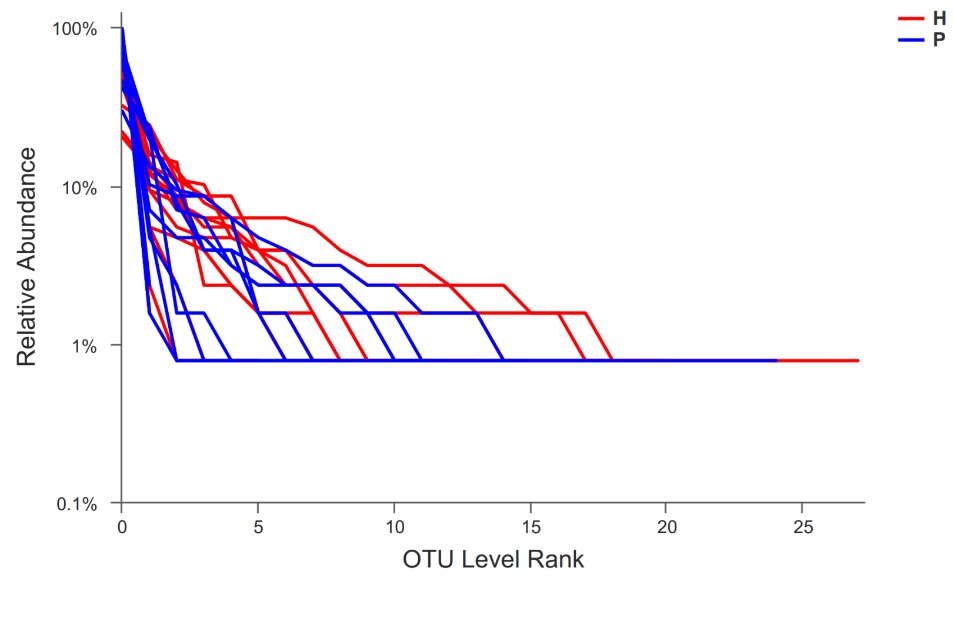

Supplement: Supplementary file 1 — Additional file 1: Figure S1. Rank-abundance curve. The horizontal coordinate represents the OTU number ranking, the vertical coordinate represents the relative percentage of species, and the horizontal coordinate at the end of the curve extension represents the number of species in the sample (the blue line represents the PJS patient group; the red line represents the healthy control group). [file 13099_2024_617_MOESM1_ESM.jpg]

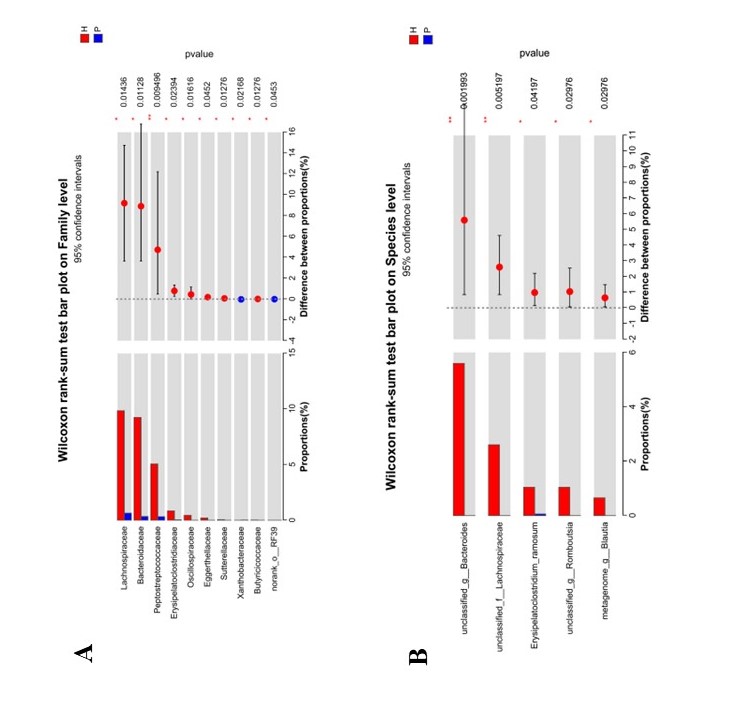

Supplement: Supplementary file 2 — Additional file 2: Figure S2.Differences in family and species abundance between groups. Different colors represent different groups, and the X-axis represents the average relative abundance of different groups and the differences between groups at the family and species levels (* P<0.05, ** P<0.01; H: the healthy control group, P: the PJS patient group). [file 13099_2024_617_MOESM2_ESM.jpg]

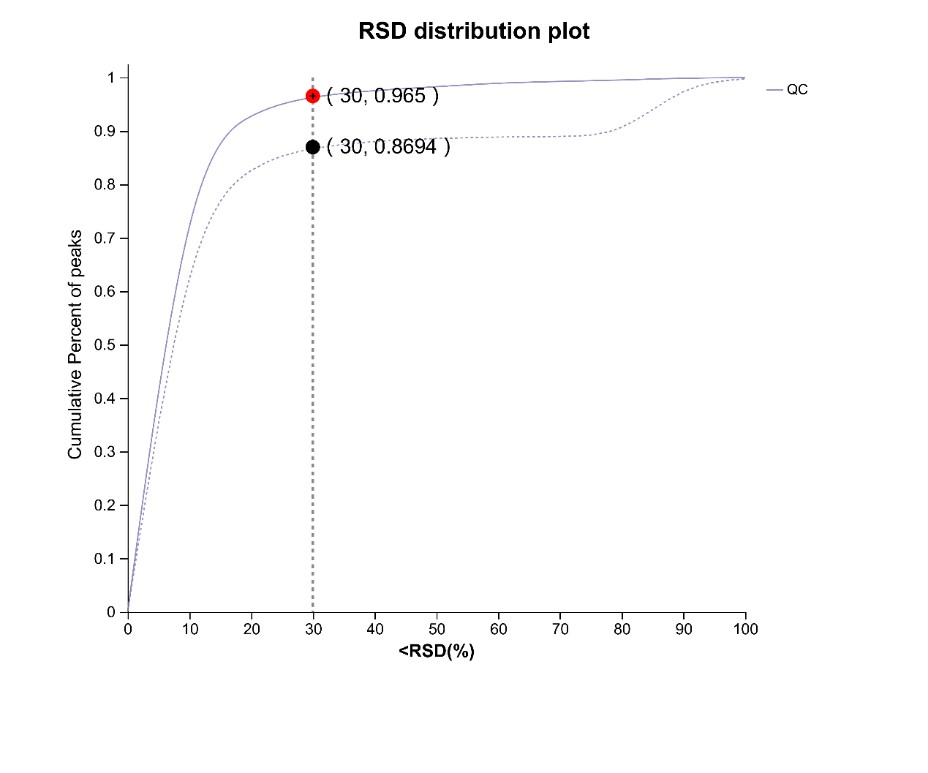

Supplement: Supplementary file 3 — Additional file 3: Figure S3. Relative standard deviation (RSD) distribution curve. For the overall data, RSD<0.3, and the cumulative proportion of peaks<70%, suggesting that the quality control data are qualified and can be further analyzed. [file 13099_2024_617_MOESM3_ESM.jpg]

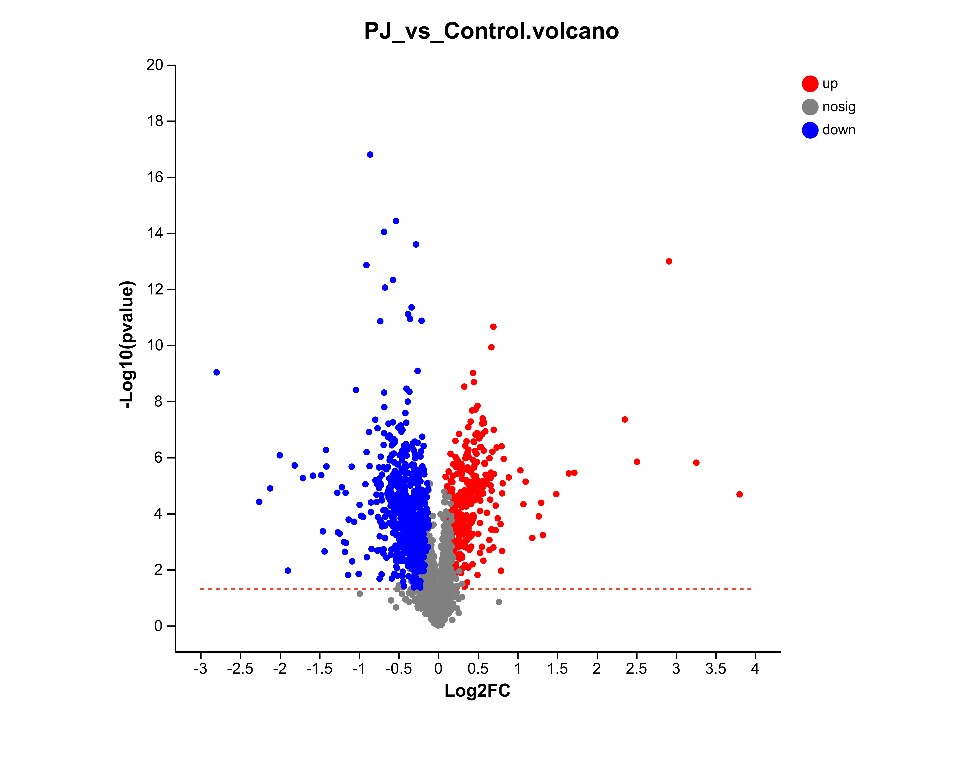

Supplement: Supplementary file 4 — Additional file 4: Figure S4. Volcanic map of differences between anions and cations. The red dots represent metabolites with upregulated expression, the blue dots represent metabolites with downregulated expression, and the gray dots represent metabolites with no significant differences. The horizontal coordinate is the change value of the difference in the expression of metabolites between the two groups, log 2FC (fold change value); the vertical coordinate is the statistical test value of the difference in the expression of metabolites. [file 13099_2024_617_MOESM4_ESM.jpg]
